# Soil transmitted helminths and *schistosoma mansoni *infections among school children in zarima town, northwest Ethiopia

**DOI:** 10.1186/1471-2334-11-189

**Published:** 2011-07-09

**Authors:** Abebe Alemu, Asmamaw Atnafu, Zelalem Addis, Yitayal Shiferaw, Takele Teklu, Biniam Mathewos, Wubet Birhan, Simon Gebretsadik, Baye Gelaw

**Affiliations:** 1 Department of Medical Laboratory Sciences, College of Medicine and Health Sciences, University of Gondar, Gondar, Ethiopia

## Abstract

**Background:**

In Ethiopia, because of low quality drinking water supply and latrine coverage, helminths infections are the second most predominant causes of outpatient morbidity. Indeed, there is a scarcity of information on the prevalence of soil transmitted helminths and Schistosomiasis in Ethiopia, special in study area. Therefore, the aim of this study was to determine the prevalence and associated risk factors of soil transmitted helminths and intestinal Schistosomiasis.

**Methods:**

Cross-sectional study was conducted among 319 school children of Zarima town from April 1 to May 25, 2009. A pre-tested structured questionnaire was used to collect socio-demographic data and possible risk factors exposure. Early morning stool samples were collected and a Kato Katz semi concentration technique was used to examine and count parasitic load by compound light microscope. Data entry and analysis was done using SPSS-15 version and p-value < 0.05 considered statistically significant.

**Results:**

Out of 319 study subjects, 263 (82.4%) of the study participants infected with one or more parasites. From soil transmitted helminths, *Ascaris lumbricoides *was the predominant isolate (22%) followed by Hookworms (19%) and *Trichuris trichiura *(2.5%). *Schistosoma mansoni *was also isolated in 37.9% of the study participants. Hookworm and *S. mansoni infections *showed *s*tatistically significant associations with shoe wearing and swimming habit of school children, respectively.

**Conclusion:**

Prevalence of soil transmitted helminths (STH) and *S.mansoni *was high and the diseases were still major health problem in the study area which alerts public health intervention as soon as possible.

## Background

Soil transmitted helminthic infections and Schistosomiasis are among the widely spread chronic infections in the world. Globally 2 billion individuals are infected with helminths, out of these majorities live in resource-poor settings [1,2]. World health organization (WHO) estimated the common STHIs in world as: 250 million cases for Ascariasis, 151 million cases of hookworm diseases, 100 million cases of Strongyloidiasis and 45.5 million cases of Trichuriasis [3]. Schistosomiasis is also remains one of the most prevalent parasitic diseases in the world. It is endemic in 76 countries and continues to be public health concern in developing countries. Approximately 80% of the 200 million people infected world-wide live in sub-Saharan Africa where *Schistosoma mansoni *and *Schistosoma haematobium *are widespread [4,5].

The occurrence of helminthic infections is associated with socioeconomic, environmental and other factors like, ignorance of simple health promoting factors and overcrowding, limited access to clean water, tropical climate and low altitude [2]. School age children are one of the groups at high-risk for intestinal parasitic infections. Factors like poor developments of hygienic habits, immune system and over-crowding contributes for infection [6]. The adverse effects of intestinal parasites among children are diverse and alarming. Intestinal parasitic infections have detrimental effects on the survival, appetite, growth and physical fatnesses, school attendance and cognitive performance of school age children [7-11].

Ethiopia has one of the lowest quality drinking water supply and latrine coverage in the world [12]. Because of this and other reasons intestinal parasites have been widespread in Ethiopia and parasitic helminthic infections are the second most predominant causes of outpatient morbidity in the country. Many reports illustrated that Ascariasis is the most prevalent intestinal parasitic infection in different communities usually occurring together with trichuriasis. Hookworm infection, Strongyloidiasis and Enterobiasis are also public health problem though the magnitude is lesser compared to Ascariasis [13]. Also schistosomiasis is common in northern region as compared to south and south west regions of Ethiopia [14].

Intervention against STH and *Schistosoma *infections are based on regular anti-helminthic treatment, improved water supply, sanitation and health education [15-17]. STH infections have not been targeted for control in Ethiopia [18], though mass de-worming as a component of the Enhanced Outreach Strategy (EOS) targeting under five children started in 2004 [19]. Low-cost, high-coverage delivery of anti-helminthic treatment has been achieved in some settings [20] but improving sanitation is more complex. In Ethiopia, for example, levels of access to improved sanitation in rural areas are very low (5.4%) making evaluation of other components of intervention important [21].

Prevalence of intestinal helminths and other intestinal parasites have been studied in different of tropics and subtropics including Ethiopia [22-32]. Although many studies previously conducted in Ethiopia, gave due attention to distribution of different intestinal parasites on different altitudes in different community groups such as preschool children, school children and other groups confined to camps and refugees, the prevalence of STH and schistosomiasis was not well addressed indifferent parts of Ethiopia including our study area. Therefore, the aim of this study was to determine the prevalence and associated risk factors of soil transmitted helminths and *Schistosoma mansoni *infection among school children in Zarima town, North Gondar, North West Ethiopia. As result this study envisages that it might strengthen the information so far for scaling up and to design effective communication strategy to combat STH and *S. mansoni *in the study area.

## Methods

### Study area

Cross-sectional study was conducted from April 1 to May 25, 2009 among elementary school children of Zarima town which is one of small town in "Adarkay" woreda in North Gondar. It is 140 km far from Gondar town. The town has approximately a total population of about 20, 000 [33]. The area is predominately rural and most residents live in villages as agriculturists which is mixed agriculture; growing maize, teff (Eragrostos teff) and wheat. The altitude of the town range from 1000-2400 meter above see level and the topography shows hills and plain land with springs, streams and rivers which are often source of water for domestic and other uses for the communities in the town and around the town. These predispose the children to water borne diseases during swimming, washing, playing, and crossing the water. The town has only one government owned general elementary school which encompasses 25 teaching sections within a single compound.

### Sample size determination and sampling techniques

The sample size for this study was calculated by using single proportion formula at 95% confidence interval (CI) level (Z (1-ά/2) = 1.96), an expected prevalence of 50% since there was no study conducted regarding this topic in the area and 5% marginal error. Then the sample size was calculated as n = [Z *1- α/2*] 2 *P (1-p]/d^2^*, Where: n = sample size, P = proportion problem in the study area, Z 1-α/2 = CI of 95%, d = Marginal error to be tolerated. By adding 10% of contingency 319 students were included in our study.

The sample size which is determined earlier was used to calculate the proportion of students to be selected from each section. Using registration list, simple random sampling method was employed to select students from each section using a table of random numbers and when the selected student was absent student before or after the indicated one was sampled for replacement.

### Socio demography and risk factor assessment

Well structured questionnaire based on the known risk factors for STHs and Schistosoma was locally developed specifically in English version by the research team. It was later translated into the Amharic, local language of the study area. Comparisons were made on the consistency of the two versions. The questionnaires address students' socio-demographic information, hand washing, shoe wearing and swimming habit, presence of latrine and its usage, water source for domestic use and other risk factors around. A pretest was conducted among five percent of the total sample size that was selected randomly from 25 sections on which cross sectional study was conducted by trained data collectors and any ambiguous questions and repetitive ideas were corrected. Additional response categories were also added based on the pretest findings. When the children were identified, the purpose, objectives and benefits of the study were explained to the parents and consent for participation sought. Those who consented were interviewed at the school. All households were inspected to assess the type and condition of latrine, status of the compound, absence or presence of refuse dumps, soiling of the compound and whether the home reared cattle. The type of water sources from which respondents collected water and their accessibility was also noted.

### Stool sample collection, processing and light microscope detection

After interviewed the students were then given labeled stool containers with tight covers bearing serial numbers of the subjects and were asked to put about 5 g of stool the following morning. All the stool samples were received the following day at an organized central place and processed using direct saline wet mount for motile intestinal parasites. Stool samples also processed using Kato-Katz smear technique [34]. The slides were examined under light microscope at X100 and X400 magnifications. The parts of the slides was re-examined for assurance of quality control. A small portion of the stool samples were also preserved in 10% formalin for repeating the tests whenever required.

### Quality control

For quality purpose Proper thin wet mount and Kato-Katz techniques was prepared using standard amount of specimen and examined within a given time. Ten percent of the slides was randomly selected and re-examined at the end by experienced laboratory technologist who was blind for the first examination result. The result of laboratory examination was recorded on well prepared format carefully and finally it was attached with the questionnaire.

### Data management and analysis

After data collection process, the data were checked for completeness. Then the result of laboratory examination was recorded on well prepared format carefully and finally was attached with the questionnaire. The study population was further divided into three age groups, i.e. 5-9, 10-14 and 15-19 years. The egg per gram stool (EPG) was used to categorize intensity of infection. The intensity of infection was classified as low (when EPG was < 200), moderate (EPG = 201-800), and heavy (EPG > 800). The frequency distribution of both dependent and independent variables were worked. The dependent variables were any STH and Schistosoma infections in the children. Potential risk factors explored include sociodemographic characteristics, hand washing, shoe wearing and swimming habits, presence of latrine and its usage and others. The data was entered into a computer using SPSS (15 version) statistical packages and univariate association between each exposure and the presence of infection was assessed using the Chi-squared test and odds ratios with 95% CI were computed as measures of association. P-value less than 0.05 were taken as statistical significant association between dependent and independent variables.

### Ethical consideration

Ethical clearance was obtained from University of Gondar, College of medicine and health sciences and department of medical laboratory sciences. Additionally, after explaining the importance of the study briefly an informed written consent was obtained from Parents of the study subjects. Anyone not willing to take part in the study had full right to do so and confidentiality of the study participants was also maintained. Those students who were positive for intestinal parasites were treated accordingly by appropriate antiparasitic drugs.

## Results

### Socio-demographic characteristics of study subjects

A total of 319 school children (157 males and 162 females) were included in the study. The age range of interviewed children was 5 to 17 years with median of 11 (± 1.2sd) years. The majority 89.4% and 85.9% of students were Amahara by ethnicity and Orthodox Christians by religion, respectively. 70.8% of student's family heads were illiterate and agriculture was their major occupation (76.4%).

### Prevalence and intensity of soil transmitted helminths and *Schistosoma mansoni*

Out of 319 study subjects 263 (82.4%) and 251(78.1%) had one or more parasites in Kato Katz and wet mount techniques, respectively. From soil transmitted helminths, *A. lumbricoides *was the predominant isolate (22%) followed byhookworms (19%) and *T. trichiura *(2.5%). *S. mansoni *was also isolated in 37.9% of the study participants. Though statistically significant difference was not observed in parasitic infection among males and females but infections due to *A. lumbricoides *and Hookworms appeared relatively higher in males than in females. On the other hand more females than males were affected by *T. trichiura *and *S.mansoni *(Table 1).

**Table 1:** Prevalence of STH and *S. manson i *in Children Attending Zarima elementary School, 2009

Parasite s indentified	Male (N = 157)		Female (N = 162)		Total (N = 319)		X^2^	P.value
	No	%	No	%	No	%		
*S. mansoni*	53	33.7	68	42.2	121	37.9	2.28	0.06
*A.Lumbricoides*	49	31.2	21	12.9	70	21.9	15.5	0.000008
Hookworms	30	19.1	30	18.5	60	18.8	0.018	0.8928
*T.trichura*	2	1.2	6	3.7	8	2.5	1.925	0.1654
Others	3	1.9	1	0.6	4	1.2	1.077	0.0301
Total	137	87.2	126	77.7	263	82.4		

More than half (53.9%)of children had a single infection, while 23.2% of children had double infections and 5.3% had triple infections. From the infected children, 45.5%, 43.7% and 10.8% harbored single, double and triple parasites, respectively. Higher rate (10.1%) of *S. mansoni *co-infection with *A. lumbricoides *was observed followed by hookworms (7.2%) and *T. trichiura *(0.9%). There was no statistically significant association between multiple infection and sex and age. The rate of heavy infection was highest for *A. lumbricoides *(14.1%) followed by *S. mansoni *(7.4%) and Hookworms (3.4%). In contrast no heavy infection was detected due to *T. trichiura *(Table 2). On the other hand 38.5%, 53.6% and 7.9% of the infected children harboured low, moderate and heavy infection, respectively for different STH and *S. mansoni*.

**Table 2:** Categorization of Intensity of Infection Due to *S. mansoni, A. Lumbricoides, T. trichiura *and the Hookworms in School Children in Zarima Town, 2009

Infection status	*S.mansoni*		*A.Lumbricoides*		Hookworms		*T. trichiura*	
	No	%	No	%	No	%	No	%
Negative	198	62.1	193	60.5	203	63.6	311	97.5
Low	32	10	20	6.3	39	12.2	6	1.9
Moderate	80	25	40	12.5	19	6	2	0.6
Heavy	9	2.9	10	3.1	2	0.6	0	0
Total positive	121 8	37.9	70	21**.9**	60	18.8	8	2.5
Total	319	100	319	100	319	100	319	100

### Univariate risk factor analysis for soil transmitted helminths and *S. mansoni *infections

The distributions of the demographic and risk factors of the subjects are shown in Table 3. In univariate analyses, no statistical significant association were observed in parasite prevalence between male versus female, first versus second cycle and maternal educational status of the students. Even though there was no overall statistical significant association between age and parasite prevalence; age group between 5-9 years showed statistical significant association compared to other categories (OR = 3.08; 95% CI 1.017, 9.373, p = 0.047). Similarly significant association in parasite prevalence was observed between family water source (overall p < 0.005) with the highest prevalence was observed in those getting their water from protected spring (89.5%), and the lowest in those using pipe line (74.4%). In addition, frequency of hand washing habit with soap was significantly associated with children infection with increased risk seen for infrequent hand washing habit with soap compared to daily use (overall p = 0.000, OR 0.292, 95% CI 0.130-0.652) (Table 3).

**Table 3:** Univariate risk factor analysis for soil transmitted helminths and *S. mansoni *infections among school children in Zarima town, northwest Ethiopia, 2009

		STH and *S.mansoni *in the children (N = 319)		
Variables	N(%)	n(%)	OR (95%CI)	p-value
Demographics variables				
sex				
Male	157(49.2)	137(87.3)	0.913(0.827,1007)	0.067
Female	162(50.8)	126(77.7)		
Age categories				
5-9	47(14.7)	43(91.5)	3.08(1.017,9.373)	0.047
10-14	151(47.3)	129 (85.4)	1.68(0.904,3.134)	0.101
15-19	121(38)	91(75.2)	1	0.071
Education level				
First cycle(1-4)	160(50.2)	139(86.9)	0.650(0.329,1.093)	0.093
Second cycle(5-8)	159(49.8)	124(77.9)		
Mother's education				
Illiterate	226(70.8)	191(84.5)	0.764(0.407,1.431)	0.399
Literate	93(29.2)	72(77.4)		
Risk factors				
Water source				
Pipe line	117(36.7)	87(74.4)	1	0.005
Protected spring	153(48)	134(87.6)	0.483(0.196,1.190)	0.114
Well	49(15.3)	42(85.7)	1.427(0.556,3.701)	0.465
Hand washing with soap				
Daily	30(9.4)	10(33.3)	1	< 0.001
At least once a week	135(42.3)	112 (83)	0.311(0.011,0.086)	< 0.001
Less frequent than once a week	154(48.3)	141 (91.5)	0.292(0.130,0.652)	0.003
Latrine type				
Private toilet	132(41.4)	112(84.8)	1	0.829
Public toilet	30(9.4)	25 (83.3)	0.878(0.325,2.616)	0.878
Open field	157(49.2)	126 (80.2)	1.12(0.384,3.27)	0.836

**Note**: STH = soil transmitted helminths, N = total number of study participants, n = number of outcome for each exposure.

Separate univariate analysis for specific parasite prevalence and risk factors indicated significant positive associations were seen between presence of *Hook worm *infection and none-shoe wearing habits(OR = 2.1;95% CI 1.55-4.494, p < 0.001) and *S. mansoni *infection and those children who had swimming habit in the river (OR = 3.2;95% CI 2.53-8.38, p < 0.001) (data not presented here).

## Discussion

Soil-transmitted helminths and schistosomiasis infections are among the most prevalent afflictions of humans who live in areas of poverty in the developing world. Because of the geographic overlap of these afflictions and their impact on children and adolescents, World Health Organization (WHO); the World Bank; and other United Nations agencies and bilateral, and civil society are working to integrate STH and *Schistosoma *control through a program of periodic school-based targeted Antihelminthic drug treatments [16,17].

Despite presence of the deworming program for control of STH and *S. mansoni *currently in study area, the finding this study showed that over all prevalence STH and *S. mansoni *was high (82.4%) and the diseases were still a major health problem of the study area. *S. mansoni *was most prevalent (37.9%,) followed *A. lumbricoides*, the Hookworms, *T. trichiura *and any other helminths, 22%, 19%, 2.5% and 1.5%, respectively. The finding of this study is quite high when compared to other studies conducted in Gondar regions and other parts of Ethiopia [23-26].

A cross sectional survey conducted on STH and *S.mansoni *infection among school children in Chilga district, North West Ethiopia [22] reported that the over all prevalence rate for one or multiple parasitic infections in children was 68.4% and infection due to *A. lumbricoides *was the most prevalent (42.9%, range: 22.9%-68.6%) followed by the hookworms (37.7%, range: 28.0% - 65.5 *%), S. mansoni *(19.4%, range: 7.0% - 64.3%) and *T. trichiura *infection (14.8%, range: 12.7%-20.8%). The differences in prevalence among the different communities appear to be associated with environmental sanitation, water supply and socioeconomic status of households, although this needs to be verified in more extensive follow up studies. Other factors related to macro-and micro-environment, time of study, method of examination, etc, do also contribute to the differences in the prevalence and distribution of these intestinal helminths. No significant difference was obtained in infection rates and egg counts among the age and sex of schoolchildren under consideration. This denotes a similar exposure risk to infection by these helminths.

Other study conducted on intestinal helminthic infections in school children in Adarkay District, northwest Ethiopia, [23] reported overall prevalence rates of 55.3% for *S. mansoni*, 43.0% for *A lumbricoides*, 20.2% for hookworm and 11.8% for *T. trichiura *infections. In similar other studies undertaken in school children the same author has recorded infection rates of 41.3% with 35.0%, 16.5% and 22.8% for *A. lumbricoides, S. mansoni, T. trichiura *and hookworm infections, respectively, in the Dembia Plains [24]; and 35.6%, 17.3%, 8.5% 3.3% for *A. lumbricoides, S. mansoni, T. trichiura *and hookworm, respectively, in Gondar town and surrounding areas [25-28].

When compared with the above studies which were conducted before in the study area and around the study area prevalence of these parasites are still high and being increasing in the study area. This might be due to climatic condition of the Zarima town because climate is an important determinant of transmission of these infections with adequate moisture and warm temperature essential for larval development in the soil [29,30]. Equally important determinants are poverty, inadequate water supplies and sanitation [31]. Therefore, increased prevalence of *S.mansoni *infection in study area in addition to STH was mainly due to frequent contact of school children for washing, swimming and bathing activities with Zarima River which passes the town.

In this study STH and *S.mansoni *were co-endemic in the study area. The rate of heavy infection was highest for *A. lumbricoides *(14.1%) and 14.7% of the infected children harbored heavy infection for different STH and *S. mansoni*. There is evidence that individuals with many helminthic infections have been heavier infections with STH and *S.mansoni *because morbidity from these infections and rate of transmission are directly related to the numbers of worms harbored in the host [32], and the intensity is mainly epidemiological index used to describe soil transmitted helminthic infection. Also the most striking epidemiological features of human helminthic infections are aggregated distributions in human communities, predisposition of individuals to heavy (or light) infection, rapid re-infection following chemotherapy, and age intensity profiles that are typically convex (with the exception of hookworm).

The high prevalence rate of STH and S. mansoni infections encountered among schoolchildren of the study area raises a serious concern. It signifies the fact that children are the highest risk groups in the community and serve as sources of infection and transmission. These parasites are well known to be associated with lowered work capacity and productivity both in children and adults and increased susceptibility to other infections. Helminths also impair the mental and physical development of children [7,8]. Again the majority of wormy children are not only infected with one species of worm but they also tend to harbor the heaviest burdens and more than two-thirds of children were infected with one or more helminths.

Both host-specific and environmental factors have been identified that may affect the risk of acquiring or harboring heavy intensity helminthic infections. Some of factors are behavior; household clustering, occupation, poverty, sanitation and urbanization have been associated with these helminthic infections [1,2]. The result of this study revealed that age group between 5-9 showed statistical significant association compared to other categories and significant association in parasite prevalence was observed between family water sources. In addition, frequency of hand washing habit with soap was significantly associated with children infection with increased risk seen for infrequent hand washing habit with soap. This finding is almost comparable with other study findings [22,23,26]. The high infection rate among 5-9 age groups is may be due to at this age stage the students have less knowledge about sanitation and more active for swimming and playing on soil. So these factors in addition to other unknown factors might be contributed the high prevalence of STH and *S. mansoni *among 5-9 years age groups.

The strengthen of this study is that it used standard Kato Katz techniques to detect and count parasites ova, qualified laboratory technologists examined the slide in order to not miss the parasite and the questionnaire assessed many risk factors but the study only used single stool sample which may not address day-to-day and intra-stool variations of egg output that can be the limitation of the study.

## Concussions

Prevalence of STH and *S.mansoni *was quite high and the diseases were still major health problem in the study area. Because of rapid reinfection following chemotherapy, the highly effective drugs now available and the control methods used in study area have not reduced the prevalence of both schistosomiasis and soil transmitted helminths. Therefore, in future other control activities should be focused in addition to deworming program because without improved water supplies and sanitation chemotherapy approach cannot rely on for sustainable reduction in helminthic infection frequency and intensity of infection. There is also a need for community mobilization towards provision of safe and adequate water supply, latrine construction to reduce open field defecation, and health education aimed at bringing behavioral change in the district. Periodic deworming, particularly of the school-aged children with long term improvements of sanitation should be exercised.

## Competing interests

All authors declare that they have no competing financial or any other interest in relation to their work.

## Authors' contributions

AA conceived the study, undertook statistical analysis and drafted the manuscript. AA, ZA, YS, TT, BM, WB, SG AND BG initiated the study and made major contributions to the study design and statistical analysis. All authors contributed to the writing of the manuscript and approved the submitted version of the manuscript.

## Pre-publication history

The pre-publication history for this paper can be accessed here:

http://www.biomedcentral.com/1471-2334/11/189/prepub
